# The Clinical and Bacteriological Factors for Optimal Levofloxacin-Containing Triple Therapy in Second-Line *Helicobacter pylori* Eradication

**DOI:** 10.1371/journal.pone.0105822

**Published:** 2014-08-20

**Authors:** Wei-Chen Tai, Chen-Hsiang Lee, Shue-Shian Chiou, Chung-Mou Kuo, Chung-Huang Kuo, Chih-Ming Liang, Lung-Sheng Lu, Chien-Hua Chiu, Keng-Liang Wu, Yi-Chun Chiu, Tsung-Hui Hu, Seng-Kee Chuah

**Affiliations:** 1 Division of Hepatogastroenterology, Kaohsiung Chang Gung Memorial Hospital and Chang Gung University College of Medicine, Kaohsiung, Taiwan; 2 Division of Infectious Diseases, Kaohsiung Chang Gung Memorial Hospital and Chang Gung University College of Medicine, Kaohsiung, Taiwan; 3 Division of General Medicine, Department of Internal Medicine, Kaohsiung Chang Gung Memorial Hospital and Chang Gung University College of Medicine, Kaohsiung, Taiwan; Institut Pasteur Paris, France

## Abstract

Quinolone has the disadvantage of easily acquired drug resistance. It is important to prescribe it wisely for a high eradication rate. The current study aimed to determine the clinical and bacteriological factors for optimal levofloxacin-containing triple therapies in second-line *H. pylori* eradication. We enrolled a total of 158 *H. pylori*-infected patients who failed *H. pylori* eradication using the 7-day standard triple therapy (proton-pump inhibitor [PPI] twice daily, 500 mg clarithromycin twice daily, and 1 g amoxicillin twice daily). They were prescribed with either a 10-day (group A) or 14-day (group B) levofloxacin-containing triple therapy group (levofloxacin 500 mg once daily, amoxicillin 1 g twice daily, and esomeprazole 40 mg twice daily for 10 days) by their clinicians. Follow-up studies to assess treatment responses were carried out 8 weeks later. The eradication rates attained by groups A and B were 73.6% (95% confidence interval [CI] = 63.9–85.3%) and 90.5% (95% CI = 84.5–98.1%), respectively in the per protocol analysis (P = 0.008 in the per protocol analysis) and 67.1% (95% CI = 56.6–78.5%) and 84.8% (95% CI = 76.8–93.4%), respectively, in the intention-to-treat analysis (P = 0.009). The subgroup analysis revealed that *H. pylori* eradication rates for group A patients with levofloxacin-susceptible strains were 92.9% (13/14) but it dropped to 12.5% (1/8) when levofloxacin-resistant strains existed. *H. pylori* was eradicated among all the group B patients with levofloxacin-susceptible strains, but only half of patients with levofloxacin-resistant strains were successfully eradicated. In conclusion, this study confirms the effectiveness of 14-day treatment. Importantly, the results imply that 10-day treatment duration should be optimal if a culture can be performed to confirm the existence of susceptible strains. The duration of *H. pylori* eradication and levofloxacin resistance were the influencing factors for successful treatment. This study suggests that tailored levofloxacin-containing therapy should be administered only for patients with susceptible strains because it can achieve >90% success rates.

## Introduction

The Maastricht IV/Florence-Consensus Report states that after failure of a proton-pump inhibitor (PPI)-clarithromycin-containing treatment, either a bismuth-containing quadruple therapy or levofloxacin-containing triple therapy is recommended in regions of clarithromycin resistance [1]. In areas of high clarithromycin resistance, levofloxacin containing triple therapy is recommended after failure of bismuth-containing quadruple therapy. In both cases, rising rates of levofloxacin resistance should be taken into account.

Avoiding problems due to antibiotic resistance has become an important issue when deciding a second-line therapy for *H. pylori* infection [2]–[4]. Quinolone has the disadvantage of easily acquired drug resistance [5]. It is therefore an important issue to prescribe it wisely to achieve a high eradication rate. Obviously, a quinolone-containing therapy is an important salvage therapy recommended by both the Maastricht IV/Florence-Consensus Report and the second Asian Pacific Consensus Guidelines [1], [6]. In second-line *H. pylori* eradication with fluoroquinolone-based triple therapy it has been shown that neither 7 nor 10 days of therapy provides a grade B or better report card [7]. Nevertheless, 10-day fluroquinolone triple therapies are still being used as rescue therapies in many countries [8]–[10]. The other key factor to eradication success is antibiotic susceptibility [11], [12]. The current study aimed to determine the clinical and bacteriological factors for optimal levofloxacin-containing triple therapies in second-line *H. pylori* eradication. Optimal levofloxacin-containing triple therapies refer to therapeutic regimens that have 90% or greater (grade B level) and probably at 95% or greater (grade A level) to meet the existing practice criteria (13).

## Materials and Methods

### Ethics Statement

This retrospective study was approved by both the Institutional Review Board and the Ethics Committee of Chang Gung Memorial Hospital (IRB102-5044B). All patients provided their written inform consent before undergoing endoscopic interventions. None of our patients belonged to the minors/children group.

### Patients

A total of 158 *H. pylori*-infected patients who failed *H. pylori* eradication using the standard triple therapy (PPI twice daily, 500 mg clarithromycin twice daily, and 1 g amoxicillin twice daily) for 7 days were recruited from our registered files. All patients were at least 18 years of age and had received endoscope examinations that showed peptic ulcers or gastritis. The confirmation of *H. pylori* eradication failure was defined as patients with either one positive 13C-UBT or any two positive of the rapid urease test, histology and culture after first-line eradication therapy. These patients were prescribed a levofloxacin-containing triple therapy group (levofloxacin 500 mg once daily, amoxicillin 1 g twice daily, and esomeprazole 40 mg twice daily for 10 days) for either 10 or 14 days (groups A and B, respectively). According to hospital requirements, all registered patients were followed-up to assess drug compliance and adverse effects after they finished the medication regimens. These patients underwent either an endoscopy or a urea breath test 8 weeks later. We also performed a back-up urea breath test on all participants to avoid any false-negative results. Poor compliance was defined as failure to finish 80% of all medication due to adverse effects [14], [15].

### Outcomes

The primary endpoint was the successful eradication of *H. pylori*. We also analyzed antibiotic susceptibility.

#### Culture and antimicrobial resistance

One antral gastric and one corpus biopsy specimen were obtained for *H. pylori* isolation using previously described culture methods [16]. The biopsy specimens were cultured on plates containing Brucella chocolate agar with 7% sheep blood and incubated for 4–5 days under micro-aerobic conditions. The minimal inhibitory concentration (MIC) was determined by the agar dilution test. The *H. pylori* strains were tested for amoxicillin, levofloxacin, metronidazole and tetracycline susceptibility using the E-test (AB Biodisck, Solna, Sweden). *H. pylori* strains with MIC values >0.5 µg/mL, >1 µg/mL, >8 µg/mL, and 4 µg/mL were considered to be the resistant breakpoints for amoxicillin, levofloxacin, and tetracycline respectively.

#### Statistical analysis

The primary outcome variables were the eradication rate, presence of adverse events, and level of patient compliance. Using the SPSS program (Statistical Package for the Social Sciences version 18, Chicago, IL, USA), Chi-square tests with or without Yates’ correction for continuity and Fisher’s exact tests were used when appropriate to compare the major outcomes between groups. Eradication rates were analyzed by both the intent-to-treat (ITT) and per-protocol (PP) approaches. ITT analysis included all assigned patients who had taken at least one dose of the study medication. Patients whose infection status was unknown following treatment were considered treatment failures for the purposes of the ITT analysis. The PP analysis excluded patients with unknown *H. pylori* status following therapy and those with major protocol violations. A *P*-value <0.05 was considered statistically significant. To determine the independent factors that affected treatment response, the clinical and bacterial parameters were analyzed by univariate and multivariate analyses.

## Results

A total of 158 patients were enrolled (n = 79 per group). Ultimately, 7 and 5 patients were lost during follow-up in groups A and B, respectively, resulting in 72 in the PP study for group A and 74 for group B. The demographic data of the two groups are summarized in Table 1; none of the variables was significantly different between the groups.

**Table 1:** Demographic data and endoscopic appearances of the two patient groups.

Characteristics	Group A (n = 72)	Group B (n = 74)	*P-*value
Age (years) (mean ± SD)	55.5±12.1	56.3±13.2	0.703
Gender (male/female)	34/45	39/40	0.425
Smoking	7 (9.7%)	6 (8.1%)	0.732
Previous history of peptic ulcer	69 (95.8%)	72 (97.2%)	0.627
Endoscopic findings			
Gastric ulcer	28 (38.9%)	27 (36.5%)	0.765
Duodenal ulcer	11 (15.3%)	14 (18.9%)	0.559
Gastric and duodenal ulcer	6 (8.3%)	11 (14.8%)	0.219
Unspecified (including gastritis)	27 (37.5%)	22 (29.7%)	0.320

Group A: 10-day esomeprazole/amoxicillin/levofloxacin triple therapy;

Group B: 14-day esomeprazole/amoxicillin/levofloxacin triple therapy.

The eradication rates in groups A and B are detailed in Table 2. They were 73.6% (95% confidence interval [CI] = 63.9–85.3%) and 90.5% (95% CI = 84.5–98.1%), respectively, in the PP analysis (P = 0.008) and 67.1% (95% CI = 56.6–78.5%) and 84.8% (95% CI = 76.8–93.4%), respectively, in the ITT analysis (P = 0.009).

**Table 2:** Major outcomes of eradication therapy.

	Eradication rate		
	Group A	Group B	*P*-value
Intention-to-treat	67.1% (53/79)	84.8% (67/79)	0.009
Per-protocol	73.6% (53/72)	90.5% (67/74)	0.008
Adverse event	15.3% (11/72)	28.4% (21/74)	0.412
Compliance	100% (72/72)	98.6% (73/74)	0.322

Group A: 10-day esomeprazole/amoxicillin/levofloxacin triple therapy;

Group B: 14-day esomeprazole/amoxicillin/levofloxacin triple therapy.

### Adverse events and complications

The adverse event rates were 15.3% (11/72) in group A and 28.4% (21/74) in group B (Table 3). These adverse events included abdominal pain, constipation, diarrhea, dizziness, headache, nausea/vomiting, and skin rash; however, these were mild and did not markedly disturb the patients’ daily activities with the exception of one patient who complained of headache after taking the medications and stopped taking them after 1 week. Both groups had good drug compliances (100% in group A vs. 98.6% group B).

**Table 3:** Adverse events during eradication therapies.

Adverse event	Group A	Group B	*P*-value
Abdominal pain	5	6	0.792
Constipation	1	2	0.576
Diarrhea	0	3	0.084
Dizziness	4	1	0.163
Headache	4	2	0.385
Nausea/vomiting	1	2	0.576
Skin rash	0	2	0.160

Group A: 10-day esomeprazole/amoxicillin/levofloxacin triple therapy;

Group B: 14-day esomeprazole/amoxicillin/levofloxacin triple therapy.

### Antibiotic resistance

Samples from 60 patients were cultured for *H. pylori*, and the positive culture rate was 73.3% (44/60). Hence, the antibiotic resistance rates were 2.3% (1/44) for amoxicillin and 31.8% (14/44) for levofloxacin.

**Figure 1:**
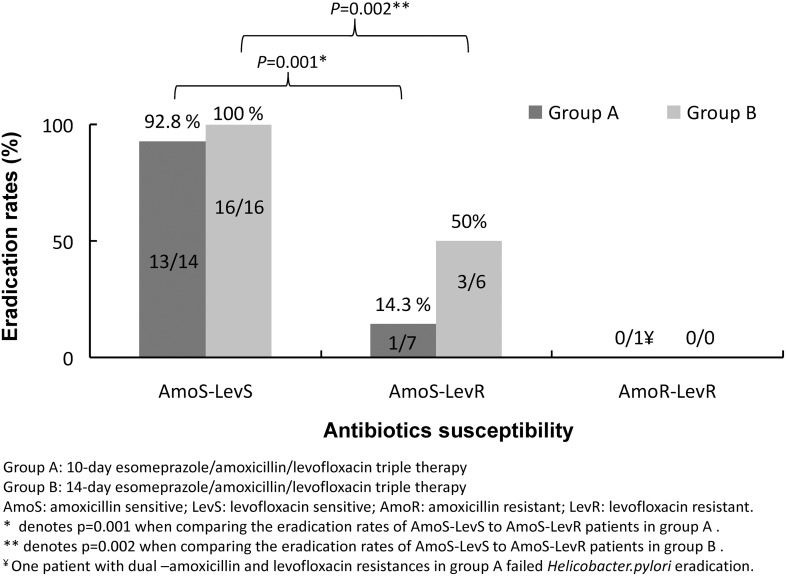
Antibiotic resistance and *H. pylori* eradication rate.

Overall, the *H. pylori* eradication rates for the levofloxacin-susceptible strains and levofloxacin-resistant strains were 96.6% (29/30) and 28.6% (4/14) in the PP analysis. Among patients with susceptible strains to both amoxicillin and levofloxacin (AmoS-LevS), 13 of the 14 patients (92.9%) in group A and all the 16 patients in group B (100%) were eradicated successfully (Figure 1). There was only one of the seven patients with amoxicillin susceptible but levofloxacin-resistant strains (AmoS-LevR) attained eradication in group A (12.5%, *P* = 0.001 compared to AmoS-LevS patients). However, three of the six patients with AmoS-LevR straints were eradicated in group B (50%, P = 0.002 compared to AmoS-LevS patients). Overall, there was only one patient with dual resistant to both amoxicillin and levofloxacin and he failed the eradication. In addition, we also observed that more patients in group B with amoxicillin-susceptible strains were successfully eradicated (86.3%) compared to group A (66.7%).

### Factors influencing the efficacy of anti-*H. pylori* therapies

Univariate analysis showed that duration of *H. pylori* eradication (*P* = 0.008), compliance (*P* = 0.031), amoxicillin resistance (*P* = 0.046), and levofloxacin resistance (*P*<0.001) were the clinical factors influencing the efficacy of *H. pylori* eradication therapy (Table 4). Multivariate analysis revealed that the duration of *H. pylori* eradication (P<0.014) and levofloxacin resistance (P<0.001) were independent risk factors for eradication failure (Table 5).

**Table 4:** Univariate analysis of the clinical factors influencing the efficacy of *H. pylori* eradication.

Principle parameter		Case no.	Eradication rate	*P*-value
Age	<60 years	75/93	86.6%	0.518
	≥60 years	45/53	84.9%	
Sex	Female	58/76	76.3%	0.053
	Male	62/70	88.6%	
Smoking	(−)	108/133	81.2%	0.318
	(+)	12/13	92.3%	
Previous history of peptic ulcer	(−)	4/5	80.0%	0.895
	(+)	116/141	82.3%	
HP eradication (per-protocol)	10-day	53/72	73.6%	0.008
	14-day	67/74	90.5%	
Compliance	Good	120/145	82.8%	0.031
	Poor	0/1	0%	
*H. pylori* culture				
Amoxicillin susceptible		43	33 (76.7%)	0.046
Resistance		1	0 (0%)	
Levofloxacin susceptible		30	29 (96.6%)	<0.001
Resistance		14	4 (28.5%)	

Group A: 10-day esomeprazole/amoxicillin/levofloxacin triple therapy;

Group B: 14-day esomeprazole/amoxicillin/levofloxacin triple therapy.

**Table 5:** Multivariate analysis of the clinical factors influencing the efficacy of *H. pylori* eradication.

Clinical factor	Coefficient	Standard error	Odds ratio (95% CI)	*P*-value
Duration of eradication (10-day vs. 14-day)	1.25	0.81	5.79 (1.29±9.50)	0.014
Levofloxacin (susceptible vs. resistance)	4.28	1.18	72.50 (7.22–727.61)	<0.001

## Discussion

The emergence of *H. pylori* strains resistant to both clarithromycin and metronidazole necessitates the identification of other rescue therapy options after initial eradication failure. Fluoroquinolones have been candidates for such rescue eradication regimens. One of these fluoroquinolones, levofloxacin, has been recommended as the second-line regimen for people who fail classical first-line therapy containing clarithromycin for *H. pylori* eradication in the Maastricht-Florence IV consensus report [1]. There are many reasons for this. Firstly, levofloxacin exerts remarkable in vitro activity against *H. pylori* [17]. Second, there is an in vitro synergistic effect of quinolone antimicrobial agents and PPIs on certain *H. pylori* strains [18]. Third, an in vitro study showed that levofloxacin retains its activity even when *H. pylori* strains are resistant to clarithromycin and metronidazole [19]. The mechanisms of action of these three antibiotics are different; therefore clarithromycin and metronidazole resistance will not influence the bactericidal activity of levofloxacin. Finally, this therapy is simple and well tolerated, with a high compliance rate (100% and 98.6% in groups A and B in the present study). Although the incidences of adverse events in groups A and B were 15.3% and 28.4%, respectively, they were mild and tolerable. Only one patient had poor compliance due to adverse events; he did not finish his medication, which resulted in eradication failure.

Generally, the eradication rate of a 10-day levofloxacin-containing regimen has been shown to be higher than that of a 7-day treatment course; therefore 10-day regimens have been widely applied as second-line therapies [9], [10]. However, studies using 7–10-day fluoroquinolone triple therapies have not been shown to obtain eradication rates >90%. Three 14-day studies that included fluoroquinolone reported eradication rates >90% were recently published [20]–[22]. The current study results further confirm the efficacy of a 14-day regimen; we attained a 90.5% PP success rate for the eradication of *H. pylori* infection.

The shortcoming of our previous report was that we did not perform antibiotics susceptibility to that patient cohort (22). In the current study cohort, sixty patients agreed to perform endoscopy for *H. pylori* culture. The novelty of this study was that we observed that even a 10-day levofloxacin-containing regimen achieved an eradication rate >90% (92.9%) against levofloxacin-susceptible strains, but there was only one patient with resistant strains who achieved eradication. This is a very important observation that suggests the identification of levofloxacin-susceptible strains before prescribing a treatment regimen. Generally, antibiotic resistance remains the key factor that is associated with either success/failure when treating *H. pylori*. Unfortunately, resistance to quinolone antimicrobial agents is easily acquired [5]. It is not surprising that the numbers of new resistant strains are increasing in most parts of the world due to plasmid-mediated horizontally transferable genes encoding quinolone resistance [23]–[27]. Therefore, we should be very careful and consider quinolone resistance when prescribing levofloxacin-containing regimens, with the end goal of achieving high eradication rates (>90%). By carefully prescribing tailored regimens according to antibiotics susceptibility, this goal can be achieved with 10-day treatment durations, as shown in the current study.

The main limitation of antibiotic sensitivity determination by Epsilometer test (E-test) is pathogen culturing, which is time consuming. However, the successful culture rate of *H. pylori* from clinical specimens is 70–80% [28], [29], so the use of other biological methods to detect antibiotic resistance in *H. pylori* is necessary and helpful. Recent breakthroughs in scientific technology allow genotypic resistance to be determined from gastric biopsy specimens with a >93% success rate, compared with the relatively lower 70% success rates of traditional susceptibility tests [30]. Resistance can also be detected by using stool samples, which means that endoscopy can be avoided [31]. Quinolone resistance in *H. pylori* is caused by point mutations (N87 and D91) in the quinolone-resistance-determining region of the *gyrA* gene [32]. The presence of a *gyrA* mutation is predictive of treatment failure with triple therapy for quinolones, such as levofloxacin [33]. In real-world practice, genotypic resistance-guided therapy is not widely commercially available to allow wide clinical application. The good news is that genotypic resistance determination for clarithromycin and levofloxacin is now commercially available (HelicoDR, Hain, Germany) [34].

Furthermore, we observed that more patients in group B with amoxicillin-susceptible strains were successfully eradicated (86.3%) compared to group A (66.7%). One possible explanation for this could be the benefit of extended use of amoxicillin to 14 days, resulting in a better outcome of PPI/amoxicillin dual therapy, which is duration and dose dependent [35]. Hsu and colleagues further explored this hypothesis and found that continuing the amoxicillin through the entire 14 days could possibly result in a Grade A *H. pylori* eradication result [36]. According to Tanimura and colleagues, the *H. pylori* eradication rate was 46.7% for 2 weeks’ treatment, increasing to 83.4% for 4 weeks’ treatment, and to 100% with 6 weeks’ treatment [35].

Amoxicillin is bactericidal and has been widely and effectively used for anti-*H. pylori* therapy worldwide; few strains are resistant to it. The antibiotic resistance rate to amoxicillin in current study was 2.3% (1/44). This patient belonged to dual resistant to both amoxicillin and levofloxacin and failed to be eradicated eventually. However, resistance to amoxicillin detected in a single strain may be asserted without taking great care. The MIC level determined by E-test might be over-estimated and misinterpreted to be resistant phenotype. There were studies, even if they have been published incorrectly, reported that *H. pylori* strains resistant to amoxicillin have never been confirmed. Only a few well-described strains with MIC verified by a dilution method were actually resistant and had mutations in the gene for penicillin binding proteins [37]. Three substitutions (Ser 414 Arg, Thr556Ser, and Asn 562) are the most common amino acid changes in penicillin binding proteins connected to amoxicillin resistance [38], [39].

## Conclusions

This study confirms the effectiveness of 14-day treatment. Importantly, the results imply that 10-day treatment duration should also be optimal if a culture can be performed to confirm the existence of susceptible strains. The duration of *H. pylori* eradication and levofloxacin resistance were the influencing factors for successful treatment. This study suggests that tailored levofloxacin-containing therapy should be administered only for patients with susceptible strains because it can achieve >90% success rates.
